# Selenium Nanocomposites in Natural Matrices as Potato Recovery Agent

**DOI:** 10.3390/ijms22094576

**Published:** 2021-04-27

**Authors:** Alla I. Perfileva, Olga A. Nozhkina, Tatjana V. Ganenko, Irina A. Graskova, Boris G. Sukhov, Alexander V. Artem’ev, Boris A. Trofimov, Konstantin V. Krutovsky

**Affiliations:** 1 Laboratory of Plant-Microbe Interactions, Siberian Institute of Plant Physiology and Biochemistry, Siberian Branch of the Russian Academy of Sciences, 664033 Irkutsk, Russia; alla.light@mail.ru (A.I.P.); smallolga@mail.ru (O.A.N.); graskova@sifibr.irk.ru (I.A.G.); 2 Laboratory of Functional Nanomaterials, A. E. Favorsky Irkutsk Institute of Chemistry, Siberian Branch of the Russian Academy of Sciences, 664033 Irkutsk, Russia; ganenko@irioch.irk.ru; 3 Laboratory of Nanoparticles, V. V. Voevodsky Institute of Chemical Kinetics and Combustion, Siberian Branch of the Russian Academy of Sciences, 630090 Novosibirsk, Russia; boris_sukhov@mail.ru; 4 A. V. Nikolaev Institute of Inorganic Chemistry, Siberian Branch of the Russian Academy of Sciences, 630090 Novosibirsk, Russia; chemisufarm@yandex.ru; 5 Laboratory of Unsaturated Heteroatomic Compounds, A. E. Favorsky Irkutsk Institute of Chemistry, Siberian Branch of the Russian Academy of Sciences, 664033 Irkutsk, Russia; boris_trofimov@irioch.irk.ru; 6 Department of Forest Genetics and Forest Tree Breeding, Faculty of Forest Sciences and Forest Ecology, Georg-August University of Göttingen, Büsgenweg 2, D-37077 Göttingen, Germany; 7 Center for Integrated Breeding Research (CiBreed), Georg-August University of Göttingen, Albrecht-Thaer-Weg 3, D-37075 Göttingen, Germany; 8 Laboratory of Population Genetics, N. I. Vavilov Institute of General Genetics, Russian Academy of Sciences, Gubkin Str. 3, 119333 Moscow, Russia; 9 Laboratory of Forest Genomics, Genome Research and Education Center, Siberian Federal University, 660036 Krasnoyarsk, Russia; 10 Department of Ecosystem Science and Management, Texas A&M University, 2138 TAMU, College Station, TX 77843-2138, USA

**Keywords:** *Clavibacter sepedonicus*, nanocomposites, phytopathogens, potatoes, selenium

## Abstract

The paper presents a study of the effect of chemically synthesized selenium nanocomposites (Se NCs) in natural polymer matrices arabinogalactan (AG) and starch (ST) on the viability of the potato ring rot pathogen *Clavibacter sepedonicus* (*Cms*), potato plants in vitro, and the soil bacterium *Rhodococcus erythropolis*. It was found that the studied Se NCs have an antibacterial effect against the phytopathogenic *Cms*, reducing its growth rate and ability to form biofilms. It was revealed that Se NC based on AG (Se/AG NC) stimulated the growth and development of potato plants in vitro as well as their root formation. At the same time, Se did not accumulate in potato tissues after the treatment of plants with Se NCs. The safety of the Se NCs was also confirmed by the absence of a negative effect on the growth and biofilm formation of the soil bacterium *R. erythropolis*. The obtained results indicate that Se NCs are promising environmentally safe agents for the protection and recovery of cultivated plants from phytopathogenic bacteria.

## 1. Introduction

Nanotechnologies have been actively introduced into various fields including agriculture [1,2,3,4,5,6]. However, despite their great potential benefit, nanocomposite (NC) materials were relatively rarely used in phytopathology, although the situation is improving, as it can be also proved by publications in this special issue.

Earlier, we studied silver and selenium (Se) NCs in natural polymer matrices as potential agents for the protection and healing of potatoes from pathogenic bacteria [7,8,9]. These substances are water-soluble, easily obtainable, and characterized by a high yield and a stable reproducible composition. In addition, NCs are of interest for their structure, which is composed of bioactive nanoparticles dispersed in a polymer matrix that is safe for living organisms and degradable by bacterial exoenzymes. It is assumed that plants treated with such NCs will not be affected themselves, while their phytopathogenic bacteria will die, and in some cases, such as in case of using NCs based on humic substances, even a positive effect on plants can be observed.

We are interested in studying effects of NCs on the Gram-positive bacterium *Clavibacter sepedonicus*, former *Clavibacter michiganense* subsp. *sepedonicum* (*Cms*) [10], a pathogenic actinomycete causing ring rot of potato, which is a devastating disease badly affecting potato yield and for which there are currently no effective means to prevent or control it [11]. We have shown earlier the antibacterial effect of silver NC in matrices of humic substances on *Cms* and the absence of negative effects on potato plants in vitro [7,9]. In the here-presented study, we tested Se NCs as a potential means of protecting and healing plants from diseases considering also their effect not only on microorganisms but also on the potato organism itself, as well as their ability to accumulate in potato tissues after treatment and to influence the microbiome inhabitants of the environment.

The main aim of the presented work was to study how Se NCs based on arabinogalactan (AG, 6.4% Se) and on starch (ST, 12.0% Se) affects the *Cms*, potato plants in vitro, and the soil microorganism *Rhodococcus erythropolis*.

## 2. Results

### 2.1. X-ray Phase Analysis (XPA) of Se NCs

**Figure 1:**
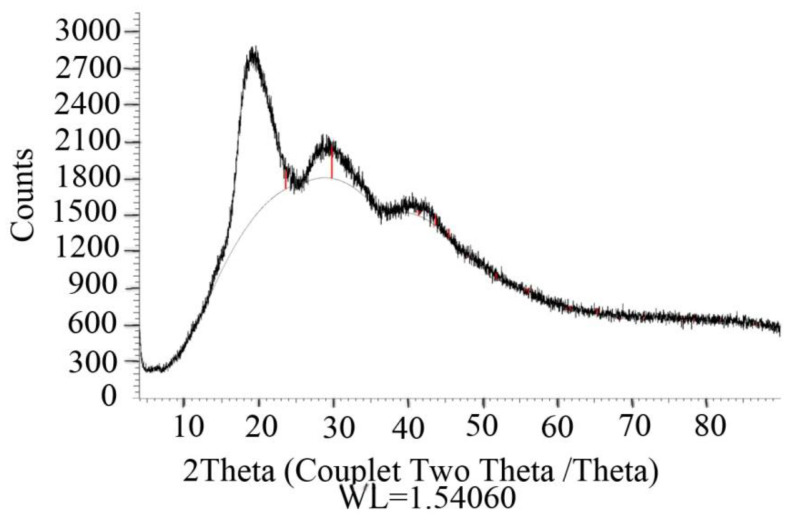
Example of a typical diffractogram of Se/AG NC based on the X-ray phase analysis (XPA).

The formation of Se NC was visually identified by the appearance of an orange-red color of the reaction mixture caused by the formation of a red modification of elemental Se [12]. The XPA pattern of the Se NC displayed a set of strongly broadened reflections, suggesting the nanoscale phase of the elemental (red) selenium [12]. While Se nanoparticles in Se/AG NC had a weakly crystalline structure with a degree of crystallinity of 1% and an average coherent scattering zone of about 2 nm calculated by the Debye–Scherer method (Figure 1), Se/ST NC was completely X-ray amorphous.

### 2.2. Transmission Electron Microscopy (TEM) of Se NCs

**Figure 2:**
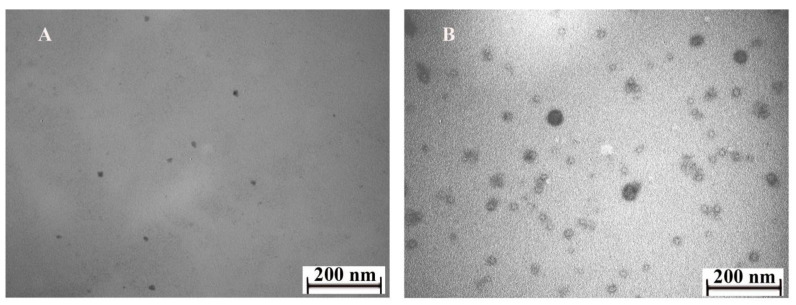
Microphotographs of Se/AG NC (**A**) and Se/ST NC (**B**) taken with a transmission electron microscope (TEM).

Using an Leo 906E transmission electron microscope (Carl Zeiss, Germany), it was found that Se nanoparticles were formed as rounded particles with average sizes of 40–60 nm for Se/AG NCs and 20–40 nm for Se/ST NCs (Figure 2). The significantly larger size (40–60 nm) of Se nanoparticles observed with TEM in Se/AG NCs compared to the average size (2 nm) of Se nanocrystallites obtained by X-ray diffraction analysis for the same NCs can be explained by a very large fraction in the material nanoparticles of an X-ray amorphous peripheral phase, which is visible through an electron microscope but whose dimensions are not determined by XPA.

### 2.3. Effect of Se NCs on Clavibacter Sepedonicus Bacteria

**Figure 3:**
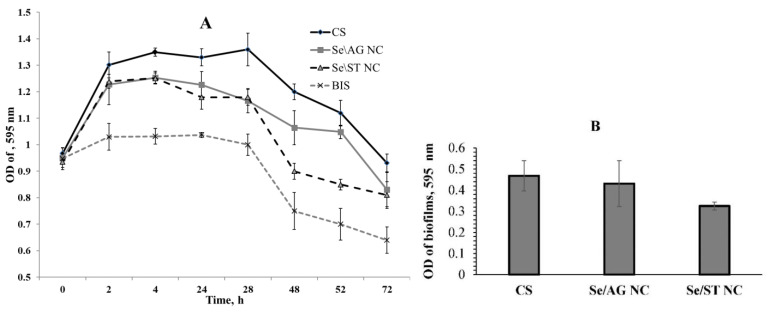
Effect of Se NC on the growth (**A**) and biofilms (**B**) of *Clavibacter sepedonicus*. OD—optical density.

In order to detect the bactericidal effect of Se NCs on potato ring rot disease, we studied their effect on bacterial growth. The results showed that a typical log curve of bacterial growth was observed in the control (Figure 3A). The BIS precursor of NCs suppressed the growth of microorganisms. Exposure to NCs was observed after 4 h of incubation with them. Se/ST NC was found to significantly inhibit *Cms* growth compared to control. The effect of Se/AG NC was less pronounced, but there was also a decrease in bacterial growth.

In addition, we investigated the effect of Se NCs on the critical ability of *Cms*-biofilm formation. It is known that this ring rot potato disease leads to the blockage of stems (wilt) [11,13], which is probably related to the formation of biofilms. Se/ST NC was found to significantly inhibit the ability of bacteria to form biofilms (Figure 3B).

We have found earlier that the morphology of the *Cms* cells changes under the influence of Se NC [8,9]. Cells are shortened and thickened, indicating adverse effects on bacteria [8,9]. At the same time, with the use of vital dyes, we have shown that almost half of the cells have died. Thus, the antibacterial effect of Se NC on the phytopathogenic bacterium *Cms* has been proven.

### 2.4. The Effect of Se NC on Potato Plants In Vitro

**Figure 4:**
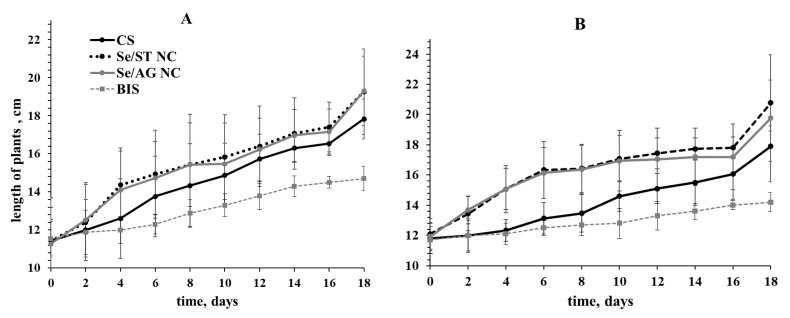
Effect of treatments by Se/AG and Se/ST NCs on the growth of Lukyanovsky (**A**) and Lugovskoy (**B**) potato varieties in vitro; CS—control samples.

The results of experiments on the influence of NCs on potato vegetation showed that the addition of NC precursor-BIS to the growth medium did not have a stimulating effect on potato growth, and in the case of Lukyanovsky variety, it even somewhat inhibited plant growth (Figure 4A). The introduction of Se/AG NC into the plant-age potato culture medium at the stage of four leafs did not adversely affect the length of the potato plants when observed for 18 days (Figure 4). Regardless of the potato variety, Se/ST and Se/AG NCs stimulated plant growth during the entire observation period (Figure 4). The effect of NCs was more pronounced in the stable potato variety Lugovskoy (Figure 4B).

**Figure 5:**
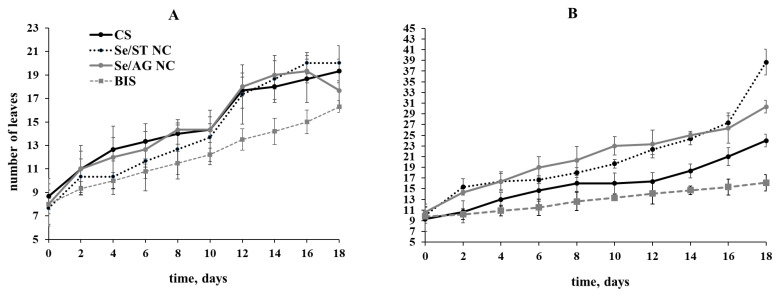
Effect of treatments by Se/AG and Se/ST NCs on the number of leaves in Lukyanovsky (**A**) and Lugovskoy (**B**) potato varieties in vitro; CS—control samples.

We also studied the effect of the Se NC treatment on the number of leaves in these potato plants (Figure 5). Analysis showed that Se/AG and Se/ST NCs slightly reduced the number of leaves in susceptible variety Lukyanovsky during the first 10 days of treatment, but further observation did not reveal any effect, the number of leaves was at the level of control (Figure 4A). In resistant variety Lugovskoy, Se NC stimulated the formation of leaves throughout the entire period of observation (Figure 5B). The treatment of plants containing BIS precursor in their media resulted in a decrease in the number of leaves in both varieties throughout the observation period. The plants did not extend in length (Table 1).

At the end of the experiment, we estimated the biomass of the above-ground part of plants and their roots (Table 1). It was found that the Se/AG NC stimulated the formation of roots in the susceptible variety Lukyanovsky compared to control. Se/ST NC increased the underground biomass of the plants of the resistant potato variety Lugovskoy.

The treatment of plants with BIS, a precursor of NC, negatively affected all plant performance. However, NCs leveled such negative effects of BIS, so Se/AG NC stimulated root formation in potatoes of the susceptible Lukyanovsky variety compared to control, and NC Se/ST increased the underground biomass of potatoes of the stable Lugovskoy variety.

In order to detect the effect of stress in plants during their treatment with Se/AG NC, a change in the activity of one of the most indicative stress enzyme, peroxidase, which actively interacts with reactive oxygen species was investigated in potato leaf tissues. Table 1 shows the results of peroxidase activity under the influence of infection and NC treatment. BIS was found to reduce enzyme activity in both varieties compared to control plants. The activity of the enzyme in plants of the susceptible Lukyanovsky variety did not change significantly after the treatment by NC. Peroxidase activity increased in the tissues of plants of the stable Lugovskoy variety.

### 2.5. EDXMA of Se and Six Other Elements in Potato Plant Samples

**Figure 6:**
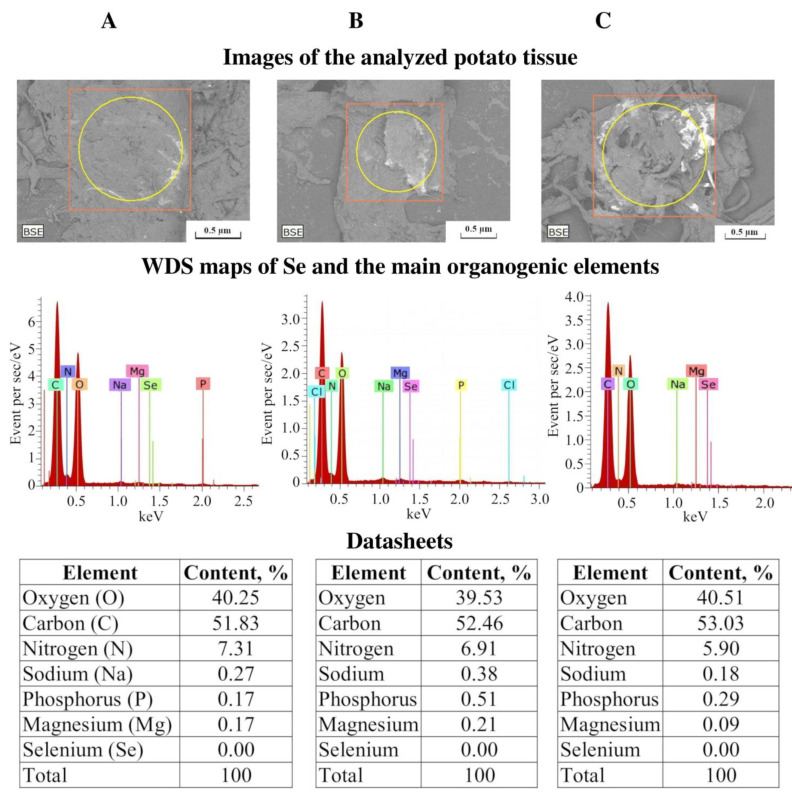
Results of energy-dispersive X-ray spectroscopy microanalysis (EDXMA) of infected *Cms* potato plant tissues (variety Lukyanovsky) untreated (**A**) and treated with Se/ST (**B**) or Se/AG (**C**) NCs. WDS—Wavelength-Dispersive Spectroscopy. Shown as an example of typical data from one of the experiments.

We checked the accumulation of Se in the potato tissues after two-day treatment by NCs using EDXMA and a Hitachi TM 3000 scanning electron microscope equipped with an Xflash 4304 SD detector for imaging (Figure 6).

In addition to Se, the presence and quantity of the following biogenic elements were also measured in the potato tissues: oxygen, carbon, nitrogen, sodium, phosphorus, and magnesium. They were not accumulated in plants treated by Se/AG and Se/ST NCs. Some, but mostly insignificant redistribution of the content of the studied elements was revealed in the samples from both varieties (Figure 6). No significant difference was found in the oxygen content in the potato tissues of the studied samples. The amount of carbon increased with the addition of NC, which was apparently due to the fact that NC were created on the basis of polysaccharide matrices, AG [(C_5_H_8_O_4_)(C_6_H_10_O_5_)_6_]_n_ and ST (C_6_H_10_O_5_)_n_, which contain many carbon molecules. The nitrogen content in the tissues of potatoes treated with Se/AG and Se/ST NCs decreased compared to the control. When Se/AG NC was used, the amount of phosphorus in potato tissues increased, which is associated with the nature of the origin of nanocomposites, the precursor of which was BIS, which contained a large number of phosphorus molecules. Similarly, an increase in the amount of sodium in potato tissues treated with Se/ST NC can be explained. The amount of magnesium in potato tissues varied depending on the NC. When treated with Se/ST NC, it slightly increased, while with Se/AG NC, it decreased.

The Se content of plant air-dry weight was very low in general in the samples from both varieties, below 0.00% (Figure 6), probably, even below the detection limit of the method. This suggests that treatment by Se/AG and Se/ST NCs are safe for all plants.

### 2.6. The Effect of Se NC on the Viability of the Soil Bacterium Rhodococcus erythropolis

An important aspect of the application of NCs in real practice is their safety for the environment and soil microbiome. To test it, we checked the impact of Se/ST and Se/AG NCs on growth and biofilm formation of soil bacteria *R. erythropolis*, which is a typical representative of soil microbiome in Siberia.

**Figure 7:**
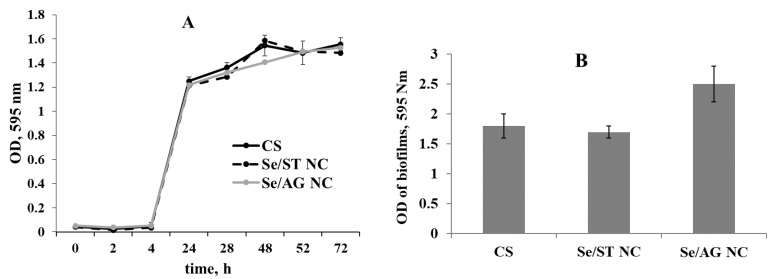
Effect of the Se NC treatments on growth (**A**) and biofilm formation (**B**) of *Rhodococcus erythropolis*. OD—optical density.

In previous experiments, the negative influence of the precursor of nanocomposites BIS on the living objects used also in this study was revealed, but BIS itself is not intended to be used in pure form for plant treatment. Therefore, in a series of tests with *R. erythropolis*, BIS was not used. The growth curve was not different from the control (Figure 7A). The method of diffusion in agar also did not detect the bactericidal effect of the studied NCs on the *R. erythropolis*.

No significant effect of NC on the ability of bacteria to form biofilms was revealed (Figure 7B), demonstrating the lack of negative effect on the biofilm formation of *Rhodococcus*.

## 3. Discussion

There are various methods of obtaining Se NCs: physical and chemical synthesis, as well as using different organisms (bacteria, algae, fungi, plants), which is an example of the so-called "green chemistry". The chemical synthesis of Se NCs is attractive due to the fast rate of obtaining the finished product, simplicity, and cost effectiveness. It is carried out by combining various chemicals. Both organic and inorganic ingredients are used as precursors. Se NCs can be obtained on the basis of various acids: polymethacrylic acid, in the process of reduction of selenous acid with succinic acid in the presence of polyvinylpyrrolidone, and on the basis of acetic, oxalic, and gallic acids [13]. Depending on the mass ratio of the initial ingredients, nanostructures of different sizes and morphology (spheres and micelles of irregular shape containing nuclei) can be formed. The nature of the polysaccharide is a determining factor in the formation of Se NCs and optimization of their parameters. For example, in a chemical synthesis, Se NCs can be synthesized using sodium borohydride as a reducing agent and gum as a stabilizer. The size of such NCs can vary from 44 to 200 nm, with the average size of ≈106 nm. It was shown that such NCs exhibited high radial absorption activity [14].

Chemically synthesized NCs can be packed in the process of synthesis into polymer molecules, in particular, into polysaccharides [15,16,17,18]. Hybrid nanosystems based on Se NCs can be synthesized by the method of chemical reduction using various stabilizers and reducing agents: bovine serum albumin (BSA) + ascorbic acid, chitosan + ascorbic acid, and glucose. It has been shown that nanocompounds obtained under various synthesis conditions and using various stabilizers and reducing agents exhibit various antimicrobial activities, as well as cytotoxicity, which are of key importance for their application [19].

The NCs synthesized in our study were evaluated by the traditional methods used for such studies; these are TEM and elemental analysis [20]. In addition, XPA was used to characterize the NCs, which has high accuracy and does not affect stability of prepared samples. This method is used not only for the study of chemical objects including Se-containing samples [21], but also for various purposes in geology [22], biology [23], and medicine [24]. XPA is also attractive because its ability to detect structures that are invisible with X-ray absorption imaging.

Potato ring rot is caused by the Gram-positive bacteria *Cms*, which is a pathogen in many countries in Europe, the USA, and Canada [25]. Damage from the disease can reach 40%, but there is currently no effective way to control *Cms*. In earlier studies, we demonstrated the bactericidal effect of the Se/AG NC with different levels of Se on *Cms* [9]. For instance, Se/AG NC with 1.23% of Se decreased the survival of *Cms* by 20% compared to the control after an hour incubation of bacteria with it. The bacterial cells looked deformed (thickened and shortened) after 24 h of incubation [9]. Spherical particles corresponding in size to the Se nanoparticles were observed attached to their surface [8,9]. Treatment of *Cms* by Se/AG NC with higher content of Se (3.4%) showed attachment of the Se nanoparticles to the surface of bacterial cells leading to the death of the bacteria, which probably was associated with disruption of the cell membrane function.

It was shown in our study that Se/AG NC (6.4% of Se) and Se/ST NC (12.0% of Se) reduced the growth of *Cms* bacteria in comparison with the control. We believe that the Se NCs had the bactericidal effect on *Cms*, which was accompanied by a change of the fatty acid composition in the bacterial cells and inhibited biofilm formation (unpublished data). Thus, we have shown that all studied Se NCs inhibit the viability of the dangerous phytopathogenic Gram-positive bacteria *Cms*. Negative effects of Se nanoparticles were also described earlier on other bacteria *Staphylococcus aureus* [19,26], but we have shown it for the first time for phytopathogens.

An especially interesting aspect of the antimicrobial activity of nanoselenium compounds, which has recently attracted attention, is the inhibition of biofilm formation in microorganisms [27,28,29,30]. Bacterial biofilm is considered as a high level of bacteria strategy in adaptation to stress [31]. With the use of Se NCs of a different composition, a significant suppression of biofilm formation of microorganisms *S. aureus* and *Candida albicans* was shown [19]. This revealed fact is extremely important, as it is known that *Cms* leads to stem wilt due to clogging of conducting vessels in potato stems [25]. Even greater effect on *Cms* was found for the Se/ST NC with high content of Se (12%), which significantly reduced the growth of bacteria and caused a significant change in the morphology of their cells [9].

However, to recommend Se NCs for practical application in controlling phytopathogens, their safety for plants and soil organisms should be well-established first. The results showed no negative effects of the Se/AG (6.4% Se) and Se/ST (12% Se) NCs on growth of potato plants. Moreover, it was found that the Se/AG NC stimulated the formation of potato roots in susceptible variety Lukyanovsky compared to control. The Se/ST NC increased the biomass of the resistant potato variety Lugovskoy. These effects of NCs seem to be related to the nanoparticles, for which it was shown in pepper *Capsicum annuum* cultivar *LJ-king* that the cultivation of pepper plants in nutrient medium with nanoparticles of iron, zinc, and copper stimulated root formation [32]. The observed effect is probably also due to the biological activity of arabinogalactan [33], which may lead to the stimulation of plant biomass growth.

It was also found that the activity of peroxidase increased in plant tissues of resistant Lugovskoy variety but not in plants of the susceptible Lukyanovsky variety. The result can be explained by the different response rate to the stress factor between different varieties: the resistant variety reacts quickly, increasing the activity of the enzyme and triggering protective mechanisms in the body of the plant, while the susceptible variety reacts to external stress factors much slower, which leads to greater susceptibility to pathogens and other stress factors.

In addition, we investigated the accumulation of Se in potato tissues after treatment plants with NCs using EDXMA. It is a widely accepted method in medical and biological research. For example, it is used in the study of drugs that carry out a targeted delivery of active substances to target cells, and it is also an important tool for the detection of nanoparticles. It is often used to improve the therapeutic characteristics of some chemotherapeutic agents. In addition, EDXMA is also used to study environmental pollution and to characterize minerals accumulated in the tissues of different organisms. It is considered a useful tool in all studies requiring the determination of endogenous or exogenous elements in different tissues, cells, or any other biological samples [34]. We have shown that Se was not found in potato tissues after treatment with Se NCs.

A key aspect of the application of Se NCs in real practice is their safety also for the environment. We have shown that Se/AG (6.4% Se) and Se/ST (12% Se) NCs were harmless for the common soil bacterium *R. erythropolis*. The Se/AG (6.4% Se) and Se/ST (12% Se) NCs had neither bacteriostatic nor bactericidal effect and did not interfere with the ability of bacteria to form biofilms. It is known that biofilm formation is an important property of bacteria that greatly helps them resist external factors [31] and is extremely important for soil microorganisms.

## 4. Materials and Methods

### 4.1. Synthesis of Selenium Nanocomposites (Se NCs)

The AG was obtained from a polysaccharide of Siberian larch (Wood Chemistry Ltd., Irkutsk, Russia). The average MW of AG was 20 kDa with a degree of polydispersion of 1.7. It was additionally purified from impurities and flavonoids by passing it through a polyamide column.

A reaction flask was filled with 1 g of AG, 0.136 g of sodium bis(2-phenylethyl)diselenophosphinate [35], and 50 mL of water. The solution was stirred on a magnetic stirrer and thermostated for 3 h at 35–40 °C. Then, 5 mL of concentrated (30%) H_2_O_2_ was added, and the reaction mixture was additionally held at the same temperature for 1 h. Isolation of the Se-containing NC and its purification from the sodium bis(2-phenylethyl)phosphinate by-product was carried out by pouring the reaction mixture into a fourfold excess of acetone or ethanol followed by washing on a filter with the same solvent.

### 4.2. Energy-Dispersive X-ray Microanalysis (EDXMA) of Se NCs and Seven Elements Including Se in Potato Plant Samples

The yield of the NCs with a Se and percentage of seven elements, oxygen, carbon, nitrogen, sodium, phosphorus, magnesium, and Se, in potato plant samples were determined based on the EDXMA data obtained using a Hitachi TM 3000 scanning electron microscope (Hitachi High-Tech America, Inc., Schaumburg, IL, USA) equipped with an Xflash 4304 SD detector. Se NCs as well as samples of crushed and slightly dried plant tissue were adhered to a microscope stage using electroconductive glue and placed into a Hitachi TM 3000 scanning electron microscope chamber, where they were subjected to electron impact. Atoms of the samples were excited by electron beam, and, thus, emitted X-rays of wavelengths characteristic of each chemical element. Analyzing the energy spectrum of X-ray emissions, we assessed the sample qualitative and quantitative composition. The resulting Se/AG NC represented a pale pink-red powder, which is readily soluble in water. The synthesis and isolation of the Se/ST NC was carried out according to the procedure described above, but ST (Sigma Aldrich, Inc., St. Louis, MO, USA) was used instead of AG. The yield of the NC with an Se content of 12.0% (based on EDXMA) was 89% (in terms of Se from its precursor). The resulting NC represented an orange-red powder that is well-soluble in water. NC solutions with the Se content of 0.000625% were used in the experiments.

To determine percentage of seven elements including Se, the plants were infected with *Cms* and treated with a Se NC solution after the plants were completely colonized by the pathogen. We used three plants per variety to prepare samples for analysis after two days of co-cultivation with Se NC solution under constant conditions of 16 h of day light and 8 h of dark at 21–22 °C. The number of repetitions for each sample was five, as well as five measurement areas in each sample; the error of determination was no more than 5%.

### 4.3. X-ray Phase Analysis (XPA) of Se NCs

XPA was used to determine the crystalline modification, degree of crystallinity, and crystallite size (mean coherent scattering region) of the Se nanoparticles. XPA of Se NCs was performed on tablets made of compressed Se NC powders using a powder diffractometer D8 ADVANCE under monochromatized Cu-Kα radiation mode Locked Coupled (Bruker Corporation, Bremen, Germany). The crystallographic phase of elemental Se in nanoparticles was identified by comparing the reflections of the experimental and reference diffractograms as described in [12].

### 4.4. Transmission Electron Microscopy (TEM) of Se NCs

TEM was used to visually determine the shape and size of the Se nanoparticles. The Se NCs dissolved in water were applied to formvar-coated grids and dried. Then, the dimensional and geometric characteristics of nanoparticles in Se NCs were studied using a Leo 906E transmission electron microscope (Carl Zeiss, Oberkochen, Baden-Württemberg, Germany) at an accelerating voltage of 80 kV. Microphotographs were taken using a MegaView II camera (Arecont Vision Costar, LLC, Glendale, CA, USA) and processed using Mega Vision photoshoot capture software version 4.0 (MegaVision, Santa Barbara, CA, USA).

### 4.5. Plant Material

Two potato varieties contrasting in resistance to pathogens were used in the in vitro experiments: resistant Lugovskoy and susceptible Lukyanovsky varieties, respectively [36]. Twenty-five plants per variety were propagated by cuttings and grown on the Murashige and Skoog growth medium (Sigma Aldrich, Inc., St. Louis, MO, USA). Potatoes were cultivated for 20 days at 26 °C and illumination of 5–6 kLk.

### 4.6. Plant Experiments

Plants were grown on the Murasige–Skug liquid nutrient medium. During the plant development phase of four leafs, an aqueous Se NC solution was added only once into the liquid culture medium with the final concentration of Se in the medium of 0.000625%. Plants were incubated for 18 days with measuring biometric traits every two days, such as length of plants, number of leaves, length of internodes, mass of roots, and vegetation parts.

Guaiacol-dependent peroxidase activity in potato leaves was determined according to the Boyarkin method [37] after three days of incubation of plants with NC.

The accumulation of Se in potato tissues was studied after treatment with Se NC using the standard technique of energy-dispersive X-ray spectroscopy microanalysis.

### 4.7. Clavibacter sepedonicus (Cms), Strain Ac-1405

*Clavibacter sepedonicus* (*Cms*) bacterial strain Ac-1405 that causes circular potato rot disease was obtained from the All-Russian Collection of Microorganisms (Pushchino, Moscow Region, Russia). It was grown on medium with glucose, peptone, and yeast extract (GPY) [38].

To study the bacteriostatic activity of Se NC on the ring rot of potatoes, the liquid culture of *Cms* was grown in the dark at 26° C on a rocker (80 rpm) in flasks containing GPY medium, pH 7.2.

### 4.8. Measuring Effect of NC on Soil Bacteria Rhodococcus erythropolis

The bacteria *Rhodococcus erythropolis* was isolated by us from the soil, endo- and risosphere of plants growing at the oil-contaminated territory near Tyreti Village in Zalarin District of Irkutsk Region, Russia. A major oil spill accident occurred in the area in 1993, resulting in the spilling of approximately 14 tons of oil. Samples of meadow soil and the following species of plants have been studied: greater burdock (*Arctium lappa*), common silverweed (*Potentilla anserina*), couch grass (*Elytrigia repens*), and acute sedge (*Carex acuta*). *R. erythropolis* was cultured for one day in the dark on a solid medium consisted of a liquid agar containing beef broth or a liquid nutrient medium of similar composition. To study the effect of NC on these bacteria, the following methods were used: antimicrobial tests by diffusion into agar, assessment of bacteriostatic activity using the optical density of the bacterial suspension, and the tablet method for determining the intensity of biofilm formation.

To determine the optical density of the bacterial suspension, *R. erythropolis* was grown on a liquid growth bacterial medium for one day. Then, aqueous solutions of Se NC with a final Se concentration of 0.000625% were added. The optical density of the bacterial suspension was measured after 0, 2, 4, 24, 28, 48, and 52 h using a tablet Bio-Rad spectrophotometer model 680 (Bio-Rad Laboratories, Inc., Hercules, CA, USA) at a wavelength of 595 nm.

The agar well diffusion method was used to study the antibacterial effect of NC in relation to *R. erythropolis*. The intensity of exposure to NC was determined by the size of the precipitation zone.

To study the effect of NC on the *R. erythropolis* biofilm formation, the culture was grown during one day in a liquid growth medium; then, the studied NC was added into the suspension and cultivated under aerated conditions for one day. Then, the optical density of the bacterial suspension (595 nm) was measured, and the suspension was transferred to 96-well polystyrene plates that were stained with 1% gentian violet at room temperature for 45 minutes after 48 h of incubation. Then, wells were washed three times with distilled water to remove unabsorbed cells. The paint from bacterial cells was extracted with alcohol, and the optical density was measured at 595 nm using a tablet Bio–Rad spectrophotometer model 680 (Bio-Rad Laboratories, Inc., Hercules, CA, USA) using the tablet method.

The experiments were carried out in three independent biological replicates. The data obtained were subjected to statistical analysis using the MS Excel statistic add-in software package.

## 5. Conclusions

In this study, two Se NCs were generated using chemical synthesis. They were water-soluble powders with Se nanoparticles in the size range of 20–60 nm. Thus, in the present work, the effect of the Se NCs in natural matrices (AG and ST) on the viability of the potato ring rot causing *Cms* bacterium, potato plants in vitro, and soil *R. erythropolis* bacterium was studied. It was found that the tested Se NCs inhibited the growth of the phytopathogen *Cms*. Se/AG NC with an increased Se content of 6.4% has a positive effect on the growth of potato plants in vitro. Se/CT NC containing 12% Se stimulated potato growth and increased the number of leaves in plants. Treatment of plants with Se NCs increased the activity of the protective enzyme peroxidase in potato leaf tissues. It has been shown that after treatment of plants with Se/AG and Se/ST NCs, Se did not accumulate in potato tissues. Thus, the studied NCs not only have a pronounced negative effect on phytopathogen but also stimulated plant growth and did not affect soil microflora. Se/AG and Se/ST NCs did not have bacteriostatic, bactericidal, and anti-biofilm effect to the common soil bacteria *R. erythropolis*. The findings make it possible to consider Se/AG and Se/CT NCs as potential environmentally friendly agents for the recovery of agricultural plants from pathogenic bacteria.

## Figures and Tables

**Table 1:** Effect of Se/AG and Se/ST NC treatments on the morphometric traits and peroxidase activity in infected potato plants in vitro.

Potato Variety	Treatment	Length of Internodes, cm	Mass of Roots, g	Mass of Vegetation Parts, g	Peroxidase Activity, U
Lukyanovsky	control	1.18 ± 0.03	0.76 ± 0.02	1.60 ± 0.18	0.129 ± 0.003
	Se/ST NC	1.27 ± 0.25	0.75 ± 0.05	1.23 ± 0.15	0.122 ± 0.010
	Se/AG NC	0.90 ± 0.10	0.88 ± 0.03 *	1.41 ± 0.50	0.073 ± 0.003 *
	BIS	0.50 ± 0.20 *	0.35 ± 0.03	0.85 ± 0.02	0.051 ± 0.001 *
Lugovskoy	control	0.70 ± 0.10	1.68 ± 0.03	1.61 ± 0.10	0.145 ± 0.005
	Se/ST NC	0.60 ± 0.30	1.70 ± 0.09	1.92 ± 0.15 *	0.189 ± 0.009 *
	Se/AG NC	0.80 ± 0.20	1.56 ± 0.06	1.65 ± 0.20	0.175 ± 0.007
	BIS	0.40 ± 0.10 *	0.90 ± 0.20 *	1.30 ± 0.30	0.123 ± 0.004 *

* Statistically different from control at *P* < 0.05 based on the Mann–Whitney nonparametric test. In each treatment, 25 samples were measured, except for peroxidase, where only three samples were measured.

## Data Availability

Data is contained within the article.
